# Participation of persons living with dementia in research: A means to address epistemic injustice

**DOI:** 10.1177/14713012241299015

**Published:** 2024-11-07

**Authors:** Ulla Halonen, Mari Aaltonen, Lina Van Aerschot, Jari Pirhonen

**Affiliations:** Faculty of Humanities and Social Sciences, 4168 University of Jyväskylä, Finland; 3837 Older people Services, Finnish Institute for Health and Welfare, Finland; Faculty of Humanities and Social Sciences, University of Jyväskylä, Finland; Faculty of Social Sciences, Tampere University, Finland; Faculty of Social Sciences, 154260 University of Helsinki, Finland; Faculty of Social Sciences (Health Sciences) and Gerontology Research Center, 7840 Tampere University, Finland

**Keywords:** dementia, participation, research, epistemic injustice, people with dementia

## Abstract

Epistemic injustice refers to wronging or mistreating individuals in terms of their capacity as knowers, based on prejudices or negative attitudes. Excluding people with dementia from research is a form of epistemic injustice. In this article, we discuss epistemic injustice associated with data collection processes and the participation of people with dementia in scientific research. The challenges of participation that we discuss pertain to the role of gatekeepers and ethical research perspectives. The arguments presented are based on previous research, experiences from our current project, and critical self-assessment regarding the latter. The aim is to shed light on what enables or prevents people living with dementia from participating in research, and how this is connected to epistemic injustice. It is known that prejudices related to dementia affect both researchers and people living with dementia: the former tend to exclude people with dementia, and the latter may practice self-silencing due to dementia-related stigma. In addition to these individual issues, we argue that epistemic injustice occurs at a structural level, where a major role is played by gatekeepers and research ethics panels. As close family members, health officials, and dementia-related associations are the main gatekeepers, their attitudes and perceptions are highlighted. In terms of ethical issues, the concept of informed consent needs to be elaborated. If the research is not expected to harm participants and may contribute to improving the lives of those with dementia, the perspective should be shifted from informed consent to ongoing consent assessment. While acknowledging the features and symptoms of dementia, researchers should be more courageous, trust in the good cause, and enable persons living with dementia to participate in research that concerns them. This is the only way for researchers to genuinely understand the social world, experiences, and needs of those with dementia and to address epistemic injustice.

## Introduction

Until recently, it was generally assumed that people with dementia could not participate in research due to difficulties in communicating their thoughts, or even a lack of thoughts (Alzheimer Europe, 2019; Bottenberg, 2022). The structures of modern society and communication can marginalize people, particularly those living with cognitive or linguistic limitations (Bottenberg, 2022; Mitchell et al., 2020). However, researchers, social and healthcare staff, and family members are increasingly recognizing the right of people with dementia to participate in research (Dewing, 2007; Grenier et al., 2024). It is only since the 2010s that dementia research has increasingly included the voices of people with dementia (Bethell et al., 2018). Although there is now a growing body of research literature that includes people with dementia as informants (Groothuijse, 2024), dementia research still faces structural challenges related to ethical and methodological issues regarding their involvement (Chandra et al., 2021).

There would appear to be significant differences between continents, and even countries, in the extent and duration of research participation by people with dementia. Research practices and policies in the United Kingdom and North America have emphasized patient and public involvement (PPI) for a longer period of time, making it more central to research ethics and practice (Bethell et al., 2018). In the rest of Europe, the inclusion of people with dementia in research has gained traction more recently, with organizations like Alzheimer Europe now actively promoting such involvement (Gove et al., 2017). In addition, the guidelines and processes adopted by different organizations and countries for the inclusion of people living with dementia in research vary (Grenier et al., 2024).

In Finland, the inclusion of people living with dementia as interviewees or respondents in research is still rare. In fact, it is rare for people living with dementia to participate in research without a partner, even outside Finland (Bethell et al., 2018). However, there is a growing consensus that the social experiences of people living with dementia cannot be fully understood without their active participation in data collection and research, making their involvement crucial for producing meaningful and ethical research outcomes. But first, some attitudinal issues need to be overcome.

## The stigma of dementia creates the basis for epistemic injustice

Although an increasing number of people are living with dementia, the disease is still highly stigmatizing (Milne, 2010; Price & Hill, 2021; Young et al., 2019). Being stigmatized entails being viewed negatively, being denied a social identity, and being isolated from mainstream society (Goffman, 1963). Past characterizations of dementia as senility continue to influence representations of the condition today (Bosco et al., 2019). Sabat (2006) has written about malignant social positioning, referring to the tendency to view people living with dementia first and foremost through their condition. Although there have been many positive and successful attempts to better include those with dementia in different spheres of society, such as developing services and building environments (Handley et al., 2017; Mitchell et al., 2003), the stigma unfortunately prevails in many ways.

Mitchell et al. (2013) analyzed how public discourses around dementia shape how it is understood and how people living with dementia are treated in research. The authors highlight that prevailing discourses can either reinforce negative stereotypes and stigma, or promote a more empathetic and equitable approach to dementia and people living with dementia. Full social inclusion involves challenging negative stereotypes and fostering a more understanding and supportive society. A serious consequence of the stigma is the *epistemic injustice* faced by those with dementia (Price & Hill, 2021; Spencer, 2023). Fricker (2007) developed the concept of epistemic injustice to conceptualize the injustice in terms of who gets their voice heard socially and societally. Fricker argues that epistemic injustice occurs when a person is denied status as a ‘knower’ because of negative presumptions that listeners have toward the speaker. Fricker proposes two further concepts. *Hermeneutical injustice* refers to situations in which a person or group faces difficulties in expressing their social experiences because the society around them is incapable of hearing or understanding their perspective. The marginalized persons or groups become ‘have nots’ regarding collective knowledge creation and distribution. *Epistemic violence* is used to describe situations where people are intentionally denied a voice in collective knowledge formation. Epistemic violence is more systemic and refers to the deliberate suppression or erasure of certain kinds of knowledge, often as a form of maintaining power over marginalized groups. In short, epistemic injustice can be seen as occurring in specific interactions, while epistemic violence is a broader, structural phenomenon of power and domination.

In recent years, the concept of epistemic injustice has been used in a wide range of fields and studies, particularly in the fields of human rights, social justice, health, and education (Kidd et al., 2017). For example, mental health patients are often not heard, or their experiences are not taken seriously in treatment decisions (Kidd et al., 2022). In particular, the experiences of people living with chronic diseases, such as fibromyalgia, chronic pain or chronic fatigue syndrome, have been studied through the concept of epistemic injustice (e.g., Heggen & Berg, 2021; Hunt et al., 2024). These studies have highlighted how patients’ subjective symptoms are often not considered credible or medically relevant, which can lead to delays in treatment or incorrect treatment decisions. Kidd and Carel (2014, 2017) have argued that hermeneutic injustice is particularly evident in clinical practice. Patients’ experiences may not be solicited, may be considered unimportant, or may even be completely marginalized in practice or research. This can lead to people living with dementia having their own voices and experiences ignored. Epistemic injustice occurs when their knowledge and experiences are not valued or taken into account, which can result in unfair treatment decisions and reinforce stigma.

The concept of epistemic injustice has recently entered the field of dementia research as well. Price and Hill (2021) argue that the stigma associated with Alzheimer’s disease is still powerful enough to result in self-silencing. Self-silencing is thus the result of a process in which an individual incorporates the prejudices and stereotypes of others into their beliefs about themselves (Luckstead & Drapalski, 2015). Self-silencing results in epistemic injustice – the loss of a voice – in an indistinguishable way. Bottenberg (2022) highlights a more straightforward form of epistemic injustice regarding people living with dementia: epistemic arrogance. This occurs when people without dementia assume that they know what it is like to live with dementia, and speak on behalf of those who have it. Speaking on behalf of people living with dementia may be well-intentioned, but nevertheless results in the loss of authentic knowledge possessed by people with the condition. Epistemic arrogance is not just about the actions of individuals, but may well be fueled by laws, policies, and assumptions that form the basis of entire social systems, be they healthcare systems or research structures (Bottenberg, 2022).

## Participation of persons living with dementia in research on dementia as an individual and societal issue

The exclusion of people living with dementia as active, knowledge-enhancing participants in academic research may be seen as a form of epistemic injustice. For a long time, dementia research was mainly conducted in the medical and health sciences, strictly from the point of view of doctors and researchers. Today, social scientists also conduct research on dementia (Novek & Wilkinson, 2019; Pinkert et al., 2021), but most of the literature is written without the direct involvement of people living with dementia themselves (Alzheimer Europe, 2019). Only in recent years has the participation of persons living with dementia gained importance in academic research (Aaltonen et al., 2021; Groothuijse, 2024; Rivett, 2017). Involving those with dementia is still likely to be perceived as complex and challenging because ethical, methodological, and epistemological questions need to be closely and critically examined (Chandra et al., 2021; Hellström et al., 2007; Sherrat et al., 2007).

In addition, dementia itself poses challenges for research participation. Conditions that cause dementia differ from other somatic diseases due to their progressive nature, complexity, and variation in symptoms. Although symptoms vary from person to person, there are some common traits. The most common symptoms of dementia are declining cognitive skills, such as thinking, orientation, perception, and learning (Cerejeira et al., 2012). Psychological and behavioral symptoms contribute to functional impairment and social challenges (Schwertner et al., 2022), and these conditions eventually have an adverse effect on a person’s communication skills (Banovic et al., 2018). For example, interviews, the most commonly used qualitative data collection method, are considered to demand high levels of logical thinking and linguistic skills from interviewees. This necessitates the adaptation of interview methods to the needs of people living with dementia (Cantley & Bowes, 2004; Groot et al., 2023). People with late-stage dementia who are no longer able to express themselves verbally are even more likely to go unheard in research, which Spencer (2023) refers to as “non-verbal testimonial injustice”. Many scholars have recently emphasized the need to develop methods to acknowledge non-verbal communication (gestures, postures, etc.,) in data collection (Bottenberg, 2022; Groot et al., 2023; Spencer, 2023).

The cognitive issues of research participants also place such high demands on research ethics evaluation that it may seem challenging to conduct the research at all. Uncertainty about whether ethical evaluation slows down the research process may also raise the threshold for involving people living with dementia in studies. It is unsurprising, therefore, that the exceptionally high standard for ethical considerations has often been cited as the reason for excluding people living with dementia from research (e.g., Chandra et al., 2021; Hellström et al., 2007). Consequently, the involvement of persons living with dementia in research must be re-evaluated on multiple levels.

In this paper, we argue that there are structural challenges in research that result in epistemic injustice regarding people living with dementia. Combining existing research literature with our own experience of involving people living with dementia, we present a theory-driven case study focusing on two main reasons for their exclusion: the role of gatekeepers and the process of applying for ethical approval (Bartlett et al., 2019; Hellström et al., 2007; Sherrat et al., 2007). By shedding light on the practical challenges encountered during data collection and how these can be overcome, we seek to encourage researchers to involve persons living with dementia in future research.

## The research setting

This article is based on our own data collection process, which formed part of a broader “Living with dementia: Social relational perspective to sustainable care”-research project. The project focuses on what it is like to live with dementia, and what kind of social support and services people living with dementia and their family caregivers need. The aim is to better understand the experiences of people living with dementia, and hence including them as informants was the only reliable way to conduct the study. In this paper, we reflect on our experiences, taking into account prior literature on the participation of people living with dementia in research.

We sought to involve people living with dementia and their family caregivers from all over Finland in our study. We also wanted to find participants from different backgrounds, for example, not just active or well-off people, who are often over-represented in studies (Alzheimer Europe, 2019). As we aimed to study older persons living with dementia (not early-onset dementia), we recruited people over the age of 68 living at home. We chose to focus on people of retirement age because the issues of daily life with dementia are very different for those who are still working age. However, the use of age as a criterion should be carefully considered to avoid inappropriately excluding people from the study (Alzheimer Europe, 2019, p. 31). When looking for interviewees living with dementia, we also sought to recruit family caregivers, particularly their adult children of working age or under 68.

Participants were recruited using various channels, with the idea that those interested in the research would contact us. The study was announced through both social media (Facebook, Instagram, and LinkedIn) and traditional media (national news and some local radio channels). We also sent emails to several senior associations. The invitation to participate was kept as short, simple, and clear as possible in an easy-to-understand style (see Alzheimer Europe, 2019, p. 58; Scottish Dementia Working Group Research Sub-Group, 2014), avoiding the use of stigmatizing language (see also Novek & Wilkinson, 2019). The same announcement was used to reach participants from both target groups. However, to reach people living with dementia, we also visited local peer groups of the Alzheimer Society of Finland and used the snowball method. In addition, we tried to reach participants through the nearest regional public provider of social and health services for the elderly, but this was not successful.

Initially, we offered participants the choice of taking part in interviews or keeping a written or audio-dictated diary. Only one person living with dementia chose to keep a diary because he could no longer speak. Based on this experience, we would like to point out that the criticism of using interviews as a data collection method (cf. Bartlett, 2012) may be misguided, as people living with dementia seemed to consider the interviews a good option. However, it is true that those with significant speaking difficulties cannot participate in interviews, and it is therefore also important to offer alternative data collection methods, which researchers have developed in recent years to address epistemic injustice (Bottenberg, 2022; Groot et al., 2023; Spencer, 2023).

As we used various channels, we did not expect to have problems recruiting a sufficient number of participants for the research, as has been reported in previous studies (Jones, 2010). We quickly obtained more than enough family caregivers to participate in the study (*N* = 46) and continued to look only for persons living with dementia. This led to some problems, which we will discuss in more detail in the next section. In the research plan, we aimed to recruit 8–12 participants who would both participate in interviews and keep a diary. Finally, 20 participants with dementia took part in the study – 19 participated in interviews and one provided a written account. The participants, aged 68–85 (average age 78), included 13 men and 7 women. Seven lived alone at home, others with their spouses, and one with her sibling. Only seven participants contacted the researchers themselves, and three of them had been encouraged by others to take part in the study.

The interviewer met all participants in their homes, either in person or online. This allowed the participants to feel safe, which is considered particularly important for people living with dementia (Tanner, 2012). Half of the interviews with respondents with dementia were conducted online, using Teams, and in some cases by telephone. An online interview was suggested when long distances would otherwise have made the interview difficult to organize, but only with participants who responded positively to the idea and were familiar and comfortable with the use of computers and electronic platforms. In some cases, the family caregiver helped the person to set up an online connection. This kind of help may be essential for people living with dementia to take part in research (Tanner, 2012).

The interviewer planned the sessions so that there was time for discussion before and after the interview, during which the participants could share their thoughts and feelings or ask questions about the research. The interviewer took notes on each interview and discussed issues related to the data by updating our Teams group immediately after the interview and also during our Teams meetings.

## Key arguments and scope of exploration

### The balance between protection and paternalism

Particularly when studying very vulnerable groups, it is paramount to adhere to the ethical principles of research in order to protect subjects from harm. At the same time, ethical guidelines should be critically reviewed to ensure that they do not unintentionally make it impossible for people living with dementia to participate in research, resulting in institutional epistemic injustice.

The critical role of ethics committees has been recognized in the Alzheimer Europe Report (2019), which raised concerns that the aim of protecting persons living with dementia from harm could lead to their exclusion from research. Grenier et al. (2024) found that in Canada, consent and participation practices vary across research institutions. These differing practices do not always adequately support the rights and participation of people living with dementia in research. We need to critically evaluate the current criteria and initiate a debate on how people living with dementia can participate in studies, ensuring high-quality research.

In Finland, the National Ethics Committee for Human Sciences has published ethical guidelines (Kohonen et al., 2019) that aim to protect vulnerable people who participate in research. According to the guidelines of the University of Jyväskylä ethics committee, an ethical review must be conducted if the research deviates from the principles of participants’ informed consent or incurs the risk of causing ‘mental harm that exceeds the limits of normal daily life.’ This was the only one of the six ethical review criteria that we considered relevant to our study. Although interviewing people living with dementia about topics such as their daily lives, care needs, and experiences of services can be regarded as sensitive (see Pesonen et al., 2011), we considered that the study would not cause the participants any harm. On the contrary, taking part in an interview study may offer respondents the opportunity to talk about their experiences of living with dementia and provide a meaningful channel for being heard.

We applied for an ethical review statement to ensure that the research project met all ethical requirements, including the possible deviation from informed consent when interviewing individuals with dementia. Even if our university ethics committee had not required a review, we would have decided to apply for one for this study to avoid future conflicts with the criteria set by scientific journals. Among the documents required for the review, we focused in particular on the ethical evaluation, where we argued for the rights of people living with dementia to make decisions for themselves and to be able to choose to participate in research that affects them. We also argued for the right of people living with memory problems to participate in research. As a result, the University of Jyväskylä ethics committee saw no obstacle to proceeding with the research as planned (ethical approval number 498/13.00.04.00/2023). Since we initiated the ethical review process six months before the study began, it did not cause any delays.

In addition to ethics committees and the processes of applying for ethical evaluation, gatekeepers also play a critical role in the inclusion of people living with dementia in research. At an individual level, gatekeepers may be representatives of organizations, caregivers or family members who have been asked by researchers to assist in contacting people living with dementia (Hellström, 2007; Singh, 2016).

People with dementia are often recruited to participate in research in collaboration with dementia-related organizations. However, there is a risk of exhausting these organizations if researchers repeatedly contact the same agencies (Doody, 2018). We approached the local social and health authorities to help us contact potential participants with dementia, but our request was passed from one professional to another, resulting in no response. Gatekeepers can play an important role in protecting persons with advanced dementia, but they may sometimes unnecessarily hinder participation (Alzheimer Europe, 2019; Hellström, 2007). Fortunately, the dementia and carer associations were largely supportive of our study and circulated the invitation to participate in our research among the members of the organizations. However, there is a risk that participants may be limited to those who are already active and engaged within these organizations. Fairness would be compromised if only active members of dementia-related organizations were invited to participate (Alzheimer Europe, 2019). To avoid this, we also approached the target group through channels other than institutions and organizations.

Gatekeepers may also serve as facilitators for participation when a person living with dementia wishes to participate, but impairments in short-term memory and executive function (Galvin & Sadowsky, 2012) prevent them from contacting the researchers. In our case, the first author visited a group for persons living with dementia, organized by the Alzheimer Society of Finland, to discuss the research and invite the group members to participate. Three individuals expressed their willingness to take part, but only one contacted the researcher afterwards. As it was possible that the other two had forgotten to follow up, rather than changed their minds, the association’s employee kindly reminded the group about the research at their next meeting. After this, one of the two again expressed interest in participating and was subsequently very pleased with his involvement.

### From informed consent to ongoing consent

Young et al. (2019) draw connections between stigma theory and epistemic injustice, highlighting how stereotypes, prejudice, and discrimination contribute to the marginalization of people living with dementia. These come into play when assessing the ability of people living with dementia to give consent to participate in a study. Chandra et al. (2021) have discussed the ethical challenges associated with dementia research in their review. The main findings showed that the key issues relate specifically to obtaining consent from people with cognitive impairment. This results in the exclusion of people living with dementia, as is often assumed.

As memory loss progresses, cognitive and language skills deteriorate, but the process is still individual. Often, the essential question revolves around the potential informant’s capacity to give informed consent. In healthcare, the Mini-Mental State Examination (MMSE) is often used to evaluate the stage of dementia, and MMSE scores have been used as a criterion for participation in research (Pesonen, 2011). If this criterion is applied, the research participants should take the MMSE test or provide documentation of testing administered by a healthcare professional.

However, requiring a document on the stage of the disease could make recruitment to the study unreasonably difficult. Medical diagnoses and records are private, and researchers are not entitled to request access to them. It would not be appropriate for researchers to administer memory tests, which require medical training. Using MMSE scores in the context of research has also been found to be problematic and harmful (Hellström et al., 2007; Pesonen, 2011) because the MMSE focuses on weaknesses. This undermines participants’ self-esteem and identity, and if people living with dementia are told that they must ‘pass the test’ in order to participate in the research, they may refuse to take the test. In addition, the result of the test is indicative. It has been argued that the MMSE does not necessarily tell us anything about a person’s ability to talk about their experiences (Bartlett & O’Connor, 2010; Hellström et al., 2007; Pratt, 2002).

Our research interest lies in the participants’ experiences and perceptions, and for this reason, it is not necessary to try to assess their exact cognitive state. As the target group comprised people living with dementia living at home, the participants were likely to be in either the early or moderate stages of dementia. We asked the interviewees to estimate their stage, but most of them did not know or remember, or it was not relevant to them. The researcher who conducted all the interviews with respondents with dementia has experience of working with people with dementia and family caregivers. Based on her experience, she assessed by observation whether the interviewee had the capacity to decide to participate in the research, that is, whether they understood what was at stake and were able to give informed consent. The interviews gave the impression that, apart from a few respondents who were clearly having more difficulty concentrating on the discussion, almost all interviewees had early or moderate dementia. Many studies on people living with dementia have only included people in the early stages of the disease. Hence, this study tells us not only about people living with mild dementia, but also about people living with moderate dementia. According to the Alzheimer Europe Report (2019, p. 59), limiting studies to the early stages of dementia cannot be justified. In our case, it would have resulted in the majority of participants being excluded from the study, and in epistemic injustice to those with moderate and severe dementia.

Cognitive decline raises the ethical question of whether a person can give informed consent to participate in research (West et al., 2017). The challenge is to ensure that a person living with dementia (or any potential participant) understands what kind of decision they are making (Doron & Werner, 2017). For example, participating in research is a very different decision from making a will. Dementia does not directly lead to a loss of legal capacity, provided that the person can make an informed decision when all necessary information is available. Legal capacity should be restricted as little as possible (Juva, 2013). As Chandra et al. (2021) point out, it should be assumed that people living with dementia have capacity unless proven otherwise. The same principle may be applied to participating in research that does not involve medical or particularly sensitive information, or pose a risk to the participant’s privacy. Dementia does not eliminate the right to self-determination. As the number of people living with dementia increases, it is crucial to note that ethical criteria or decisions cannot be based solely on the cognitive capacities of those without dementia.

The ability to give informed consent to participate in research is not the same as the ability to produce relevant information for the research (Bartlett & O’Connor, 2010; O’Connor et al., 2022). Instead of requiring informed consent, participation in research could be based on shared, supported, and proxy decision-making (Alzheimer Europe Report, 2019). In healthcare, shared decision-making refers to a practice whereby the professional discusses all options with the patient to help them make a joint decision regarding the course of treatment (Stiggelbout et al., 2015). Shared decision-making shifts the focus from who made the decision to what was decided (Daly et al., 2018; Miller et al., 2016). In supported decision-making, the decision-maker is at the center but is supported as much as possible (Alzheimer Europe, 2019; Donnelly, 2019). The support person explains the alternatives and, if necessary, interprets the signs and preferences of the individual making the decision. The support person should enable the individual to exercise their legal capacity to the greatest extent possible, in accordance with the person’s wishes (Gooding, 2013).

When it comes to consent to participate, there has been a recent move away from proxy (i.e., substituted) decision-making toward shared and supported decision-making (Stiggelbout et al., 2015). In this study, the respondent living with dementia was asked for consent at the beginning of each interview, although the interviewee or a family member had already been informed about the interview. During the interview sessions, interviewees living with dementia (*n* = 19) were given information in plain language about the study and the voluntary nature of their participation. Interviewees were asked for signed consent in face-to-face interviews and verbal consent at the beginning of the online interview recording.

In their paper, Young et al. (2019) outline a ‘process consent’ model. It is discussed as an ethical approach that ensures continuous and informed participation of individuals living with dementia in research. Process consent involves checking in with the individual on an ongoing basis to ensure that they still agree to participate in a study, or to receive a particular type of care. “Ongoing” is part of a five-part model in which relatives or staff are an essential part of the process. In recent years, the concept of ongoing consent has received increasing attention (Alzheimer Europe, 2019, p. 69) because people living with dementia may forget that they are participating in research or may not fully understand what it means to participate in the first place (Bartlett & Martin, 2002).

Sometimes the interviewer was not sure how the shared or supported decision was originally made, and who had made the decision to participate in the interview. In one case, the situation could have been interpreted as the family member making the decision on behalf of the person living with dementia:

Interviewer: Well, tell me on the tape that… yes, first tell me whether you’re involved in this voluntarily.

Person living with dementia: I guess there’s no other choice. (laughs)

Spouse carer: Yes, yes, we are. A local organization employee contacted us and asked if we would be willing to participate in something like this. And I said yes. And (spouse’s name) too.

This spousal caregiver may have made a proxy decision on behalf of the person living with dementia, although the caregiver refers to the spouse’s prior consent. Nevertheless, if the spouse had not done this, the person living with dementia might have been excluded from the study, an opportunity to participate in research and make their voice heard (see Hellström et al., 2007). When reflecting afterwards on the decision to participate, it was not clear whether the interviewee living with dementia fully understood what the research was about. However, she seemed comfortable throughout the interview. Therefore, we feel that the decision to conduct the interview was the right one and that we would do the same again in a similar situation.

Another participant forgot during the process that he wanted to participate in an interview, but eventually seemed to feel comfortable talking with the interviewer. This 84-year-old man living with dementia had already called and told the researcher that he wanted to be interviewed. But when the researcher called him later to arrange the interview, he did not remember that he had already been in touch, and the researcher had to explain what the research entailed. Finally, the interviewee was ready to meet the interviewer, showed the interviewer where to sit, and was in a good mood at the time of the interview. However, he asked questions indicating that he did not fully understand what was going on.

Interviewer: We’ve talked quite a lot about your life and everyday living. Is there anything you’d like to tell me that I haven’t already asked you about?

Person living with dementia: I just want to know what you have to ask me.

Interviewer: We don’t have any further questions than what we’ve talked about here.

Person living with dementia: How does my memory seem? Is my memory clearer, for example?

The decision to participate could be considered valid, even if it was forgotten at times and the participant seemed to confuse the interview with a memory test or a discussion with a healthcare professional. Moreover, the interviewer happened to meet the man’s daughter on the day of the interview, and she was also delighted to say that her father had happily told her he was going to participate in ‘some research.’ In addition, when the interviewer thanked the man for the interview, he replied, ‘I’m the one who wants to thank you.’ Furthermore, the body language of people living with dementia may also provide valuable information about their willingness to participate. Careful attention to the participant’s facial expressions, gestures, or posture may be interpreted as part of ensuring ongoing consent (Alzheimer Europe, 2019, p. 68; Dewing, 2007). In line with the findings of similar studies (e.g., Pesonen, 2011), the people who participated in our study seemed satisfied with their participation. Some even asked the researcher if she could come back to conduct another interview. We consequently feel that our assessment of the non-harmfulness of the study, or at least of the interview situation, was correct.

Ensuring that participants feel comfortable during the interview can be seen as part of ensuring ongoing consent (Dewing, 2007; McKeown et al., 2010). The fact that people living with dementia may not be fully aware of the purpose of the interview is not a risk if the interviewee’s privacy, integrity, and safety are not compromised. The idea of being part of something and the atmosphere during the interview might be positive experiences, despite moments of forgetfulness during the process, as with the participant above, who wanted to participate and contacted the researcher by phone. He signed a consent form at the beginning of the interview, but forgot the decisions and the purpose of the research during the interview.

A strict application of the informed consent criteria could have excluded many of our participants from the study. By excluding them, the researcher would have denied them a position as informants and experts in their own lives based on their condition. This exclusion would constitute epistemic arrogance, as Bottenberg (2022) points out, and epistemic injustice toward people living with dementia. However, the notion of ongoing consent gave a voice in the research to all those participants who had expressed their willingness to participate. As we have shown, ongoing consent is the simplest way to secure the consent of a person living with dementia during the course of research. It does not necessarily involve any relatives or staff and does not require the use of assessment methods beyond the auditory and the visual. Ongoing consent therefore differs from process consent (Young et al., 2019) in these respects.

## Conclusions

In this paper, we have discussed epistemic injustice and the participation of people living with dementia in research. Epistemic injustice refers to situations where individuals are wronged in their capacity as knowers, leading to a lack of recognition or credibility regarding the knowledge they possess (Fricker, 2007; McKinnon, 2016). Recently, there has been a crucial shift toward recognizing the rights and capabilities of people living with dementia in research participation, which is essential for producing inclusive and ethical research outcomes. We explore the ethical and methodological challenges of involving people living with dementia in research, and relate the concept of epistemic injustice to practical research. Fricker (2007) emphasizes that epistemic injustice is not only a reflection of individual prejudices, but is also structural and systemic, and it can also be called epistemic violence. We highlight the importance of overcoming barriers such as stigma, ethical challenges, and gatekeepers to ensure that people living with dementia are actively involved in research. Practical examples from the Finnish study illustrate these challenges and potential solutions.

The UK Alzheimer’s Society (2023) underscores that research is vital for improving support and care for people living with dementia. However, researchers may contribute to epistemic injustice by marginalizing the voices of those living with dementia, given their authority in setting research agendas. Identifying and addressing the factors that influence the participation of people living with dementia in research is essential to prevent the legitimization of their exclusion and to uphold equality and social inclusion (Alzheimer Europe, 2019).

Stereotypes and assumptions about cognitive abilities often contribute to the epistemic marginalization of people living with dementia, resulting in their voices being overlooked (Young et al., 2019). We contend that the ability to contribute meaningfully to research should not be determined solely by cognitive assessments, such as the MMSE, which focus on deficits rather than strengths. Ethical guidelines must be critically evaluated to prevent institutional epistemic injustice. Exclusion based on cognitive assessments alone fails to recognize the diverse capacities of individuals living with dementia. Instead, ongoing, simplified consent offers a more ethical and inclusive approach, allowing continued participation as long as individuals are comfortable and willing, regardless of cognitive fluctuations. We duly challenge the necessity of the complex process model proposed by Young et al. (2019).

This study suggests rethinking traditional research ethics to better accommodate and respect the rights and experiences of all participants, particularly those vulnerable to epistemic injustice. As highlighted by Grenier et al. (2024), inconsistencies in consent and participation practices can undermine the rights and autonomy of people living with dementia, emphasizing the need for standardized, rights-respecting approaches. Greater awareness can help reduce stigma and increase research participation among those with dementia. As McKinnon (2016) notes, knowledge is inherently social and political; hence, it is crucial that everyone is able to participate in its creation. Researchers have the power to influence research topics, so they also have the power to influence who is given a voice. As Dupuis (2012) and Clarke (2018) point out, it is important to make recommendations for other researchers and professionals who wish to involve people living with dementia in research and in the decision-making processes related to their care.

Twenty years ago, Innes and Murphy (2004), in their foundational paper on the social inclusion of people living with dementia, argued that all aspects of dementia should be mainstreamed. Two decades later, the question remains as to whether people living with dementia should be treated as a special or a different target group. Based on our shared experience, we say yes and no. Recruiting people with dementia for research requires special consideration. Social inclusion is not only about the person living with dementia, but also extends to gatekeepers, whose role has been highlighted in several previous studies (McFadyen & Rankin, 2016; Rivett, 2017; Singh, 2016; West et al., 2017). People living with dementia may be prevented from participating in research by gatekeepers at both institutional and individual levels (cf. Hellström et al., 2007).

The persistent stigma surrounding dementia marginalizes those affected and denies them a social identity. We must consider why some invited people living with dementia may decline to participate. Is it due to internalized stigma, uncertainty, or fear and mistrust? Stigma and stereotypes can influence the way in which those with dementia position themselves in epistemic practices (Young et al., 2019). Individuals living with dementia may internalize societal stigma, leading to self-silencing (Price & Hill, 2021). It is important to note that in order for people living with dementia to participate in research, specific action must be taken to achieve social inclusion, equality, and stronger rights for such individuals.

Addressing epistemic injustice in research involving people living with dementia requires the adoption of inclusive and participatory methodologies. Researchers, ethics committees, and gatekeepers must collaborate to foster a more equitable understanding of the experiences of those living with dementia. Social inclusion depends heavily on embracing differences and a willingness to celebrate diversity, not forgetting a commitment to promoting equality (Jones, 2010, p. 58).

## References

[bibr1-14713012241299015] Aaltonen M. S. Martin-Matthews A. Pulkki J. M. Eskola P. Jolanki O. H. (2021). Experiences of people with memory disorders and their spouse carers on influencing formal care: “They ask my wife questions that they should ask me”. Dementia, 20(7), 2307–2322. DOI: 10.1177/1471301221994300. 33595339 PMC8564245

[bibr2-14713012241299015] Alzheimer Europe Report . (2019). Overcoming ethical challenges affecting the involvement of persons with dementia in research: Recognizing the diversity and promoting inclusive research: Alzheimer Europe. https://www.alzheimer-europe.org/sites/default/files/2022-01/05706_Alzheimer_Europe_ethics_report_2019_92.pdf.

[bibr3-14713012241299015] Alzheimer’s Society . (2023). What we think about dementia research. Alzheimer’s Society. https://www.alzheimers.org.uk/about-us/policy-and-influencing/our-position-key-dementia-challenges/dementia-research.

[bibr4-14713012241299015] Banovic S. Zunic L. J. Sinanovic O. (2018). Communication difficulties as a result of dementia. Materia Socio Medica, 30(3), 221–224. DOI: 10.5455/msm.2018.30.221-224. 30515063 PMC6195406

[bibr5-14713012241299015] Bartlett H. Martin W. Wilkinson H. (2002). Ethical issues in dementia care research. In The perspectives of people with dementia: Research methods and motivations (pp. 47–61). Jessica Kingsley Publishers.

[bibr6-14713012241299015] Bartlett R. (2012). Modifying the diary interview method to research the lives of people with dementia. Qualitative Health Research, 22(12), 1717–1726. DOI: 10.1177/1049732312462240. 23034779

[bibr7-14713012241299015] Bartlett R. Milne R. Croucher R. (2019). Strategies to improve recruitment of people with dementia to research studies. Dementia, 18(7–8), 2494–2504. DOI: 10.1177/1471301217748503. 29327604

[bibr8-14713012241299015] Bartlett R. O’Connor D. (2010). Broadening the dementia debate: Towards social citizenship. Oxford Academic. DOI: 10.46692/9781847428585.

[bibr9-14713012241299015] Bethell J. Commisso E. Rostad H. M. Puts M. Babineau J. Grinbergs-Saull A. Wighton M. B. Hammel J. Doyle E. Nadeau S. McGilton K. S. (2018). Patient engagement in research related to dementia: A scoping review. Dementia, 17(8), 944–975. DOI: 10.1177/1471301218789292. 30373460

[bibr10-14713012241299015] Bosco A. Schneider J. Coleston-Shields D. M. Higgs P. Orrell M. (2019). The social construction of dementia: Systematic review and metacognitive model of enculturation. Maturitas, 120, 12–22. DOI: 10.1016/j.maturitas.2018.11.009. 30583759

[bibr11-14713012241299015] Bottenberg F. (2022). Epistemic arrogance, moral harm, and dementia. The Journal of Philosophy of Disability, 2, 185–208. DOI: 10.5840/jpd20226916.

[bibr12-14713012241299015] Cantley C. Bowes A. Innes A. Archibald C. Murphy C. (2004). Dementia and social inclusion: The way forward. In Dementia and social inclusion (pp. 255–271). Jessica Kingsley.

[bibr13-14713012241299015] Cerejeira J. Lagarto L. Mukaetova-Ladinska E. B. (2012). Behavioural and psychological symptoms of dementia. Frontiers in Neurology, 3, 73. DOI: 10.3389/fneur.2012.00073. 22586419 PMC3345875

[bibr14-14713012241299015] Chandra M. Harbishettar V. Sawhney H. Amanullah S. (2021). Ethical issues in dementia research. Indian Journal of Psychological Medicine, 43(5 Suppl), S25–S30. DOI: 10.1177/02537176211022224. 34732951 PMC8543611

[bibr15-14713012241299015] Clarke C. L. Wilkinson H. Watson J. Wilcockson J. Kinnaird L. Williamson T. (2018). A seat around the table: Participatory data analysis with people living with dementia. Qualitative Health Research, 28(9), 1421–1433. DOI: 10.1177/1049732318774768. 29766747

[bibr16-14713012241299015] Daly R. L. Bunn F. Goodman C. (2018). Shared decision-making for people living with dementia in extended care settings: A systematic review. BMJ Open, 8(6), Article e018977. DOI: 10.1136/bmjopen-2017-018977. PMC6009462 29886439

[bibr18-14713012241299015] Dewing J. (2007). Participatory research: A method for process consent for persons who have dementia. Dementia, 6(1), 11–25. DOI: 10.1177/1471301207075625.

[bibr19-14713012241299015] Donnelly M. (2019). Deciding in dementia: The possibilities and limits of supported decision-making. International Journal of Law and Psychiatry, 66, 101466. DOI: 10.1016/j.ijlp.2019.101466. 31706377

[bibr20-14713012241299015] Doody O. (2018). Ethical challenges in intellectual disability research. Mathews Journal of Nursing and Health Care, 1(1), 1–11.

[bibr21-14713012241299015] Doron I. I. Werner P. (2017). Alzheimer’s disease and the law: Positive and negative consequences of structural stigma and labeling in the legal system. Aging & Mental Health, 21(11), 1206–1213. DOI: 10.1080/13607863.2016.1211989. 27463564

[bibr22-14713012241299015] Dupuis S. L. Gillies J. Carson J. Whyte C. Genoe R. Loiselle L. Sadler L. (2012). Moving beyond ‘patient’ and ‘client’ approaches: Mobilising authentic partnerships in dementia care. Dementia. The International Journal of Social Research and Practice, 11(4), 428–450. DOI: 10.1177/1471301211421063.

[bibr23-14713012241299015] Fricker M. (2007). Epistemic injustice: Power and the ethics of knowing. Oxford University Press.

[bibr24-14713012241299015] Galvin J. E. Sadowsky C. H. & NINCDS-ADRDA . (2012). Practical guidelines for the recognition and diagnosis of dementia. The Journal of the American Board of Family Medicine, 25(3), 367–382. DOI: 10.3122/jabfm.2012.03.100181. 22570400

[bibr25-14713012241299015] Goffman E. (1963). Stigma: Notes on the management of spoiled identity. Simon & Schuster/Touchstone Books.

[bibr26-14713012241299015] Gooding P. (2013). Supported decision-making: A rights-based disability concept and its implications for mental health law. Psychiatry, Psychology and Law, 20(3), 431–451. DOI: 10.1080/13218719.2012.711683.

[bibr27-14713012241299015] Gove D. Diaz-Ponce A. Georges J. Moniz-Cook E. Mountain G. Chattat R. Øksnebjerg L. & European Working Group of People with Dementia . (2018). Alzheimer Europe’s position on involving people with dementia in research through PPI (patient and public involvement). Aging & Mental Health, 22(6), 723–729. DOI: 10.1080/13607863.2017.1317334. 28513210

[bibr28-14713012241299015] Grenier A. O’Connor D. James K. Imahori D. Minchopoulos D. Velev N. Mann J. (2024). Consent and inclusion of people living with dementia (PLWD) in research: Establishing a Canadian agenda for inclusive rights-based practices. Canadian Journal on Aging, 1–8. DOI: 10.1017/S0714980824000217. 38764147

[bibr30-14713012241299015] Groot B. Hendrikx A. Bendien E. Woelders S. de Kock L. Abma T. (2023). In search of epistemic justice. Dialogical reflection of researchers on situated ethics in studies with people living with language and/or cognitive impairment. Journal of Aging Studies, 66, 101154. DOI: 10.1016/j.jaging.2023.101154. 37704272

[bibr31-14713012241299015] Groothuijse J. M. van Tol L. S. Leeuwen C. C. M. van Delden J. J. M. Caljouw M. A. A. Achterberg W. P. (2024). Active involvement in scientific research of persons living with dementia and long-term care users: A systematic review of existing methods with a specific focus on good practices, facilitators and barriers of involvement. BMC Geriatrics, 24(1), 324. DOI: 10.1186/s12877-024-04877-7. 38594644 PMC11003093

[bibr32-14713012241299015] Handley M. Bunn F. Goodman C. (2017). Dementia-friendly interventions to improve the care of people living with dementia admitted to hospitals: A realist review. BMJ Open, 7(7), Article e015257. DOI: 10.1136/bmjopen-2016-015257. PMC5541590 28713073

[bibr33-14713012241299015] Heggen K. M. Berg H. (2021). Epistemic injustice in the age of evidence-based practice: The case of fibromyalgia. Humanities and Social Sciences Communications, 8(1), 235–236. DOI: 10.1057/s41599-021-00918-3.

[bibr34-14713012241299015] Hellström I. Nolan M. Nordenfelt L. Lundh U. (2007). Ethical and methodological issues in interviewing persons with dementia. Nursing Ethics, 14(5), 608–619. DOI: 10.1177/0969733007080206. 17901172

[bibr35-14713012241299015] Hunt J. Runacres J. Herron D. Sheffield D. (2024). Exploring the experience of healthcare-related epistemic injustice among people with myalgic encephalomyelitis/chronic fatigue syndrome. Qualitative Report, 29(4). DOI: 10.46743/2160-3715/2024.6519.

[bibr36-14713012241299015] Innes A. Murphy C. (2004). Introduction. In Innes C. A. Archibald C. Murphy C. (Eds.), Dementia and social inclusion: Marginalised groups and marginalised areas of dementia research, care and practice (pp. 11–17). Jessica Kingsley Publishers.

[bibr70-9147130122413] Jones M. (2010). Inclusion, social inclusion and participation. In MarciaRioux H. LeeBasser Ann Melinda Jones (Eds.), Critical Perspectives on Human Right and Disability Law. Leiden, Netherlands: Martinus Nijhoff Publishers.

[bibr38-14713012241299015] Juva K. (2013). Assessment of legal functional capacity of a neuropsychiatric patient. Duodecim; Laaketieteellinen Aikakauskirja, 129(18), 1886–1892. 24187779

[bibr39-14713012241299015] Kidd I. J. Carel H. (2014). Epistemic injustice and illness. Journal of Applied Philosophy, 34(2), 172–190. DOI: 10.1111/japp.12172. PMC5324700 28303075

[bibr40-14713012241299015] Kidd I. J. Carel H. (2017). Epistemic injustice in medicine and healthcare. In Kidd I. J. Medina J. Pohlhaus G. (Eds.), The Routledge handbook of epistemic injustice (pp. 336–346). Routledge.

[bibr41-14713012241299015] Kidd I. J. Medina J. Pohlhaus G. M. (2017). The Routledge handbook of epistemic injustice. Routledge, Taylor & Francis Group.

[bibr42-14713012241299015] Kidd I. J. Spencer L. Carel H. (2022). Epistemic injustice in psychiatric research and practice. Philosophical Psychology, 1–29. DOI: 10.1080/09515089.2022.2156333.

[bibr43-14713012241299015] Kohonen I. Kuula-Luumi A. Spoof S. K. Löfström E. Hämäläinen K. Kettunen J. Turunen R. (2019). Ihmiseen kohdistuvan tutkimuksen eettiset periaatteet ja ihmistieteiden eettinen ennakkoarviointi Suomessa. Tutkimuseettisen neuvottelukunnan ohje 2019. In Ihmiseen kohdistuvan tutkimuksen eettiset periaatteet ja ihmistieteiden eettinen ennakkoarviointi Suomessa. Tutkimuseettisen neuvottelukunnan ohje 2019. Tutkimuseettinen neuvottelukunta ja Tiedonjulkistamisen neuvottelukunta. https://www.tenk.fi/sites/tenk.fi/files/Ihmistieteiden_eettisen_ennakkoarvioinnin_ohje_2019.pdf.

[bibr44-14713012241299015] Luckstead A. Drapalski A. L. (2015). Self-stigma regarding mental illness: Definition, impact, and relationships to social stigma. Psychiatric Rehabilitation Journal, 38(2), 99–102. DOI: 10.1037/prj0000152. 26075527

[bibr45-14713012241299015] McFadyen J. Rankin J. (2016). The role of gatekeepers in research: Learning from reflexivity and reflection. GSTF Journal of Nursing and Health Care, 4(1), 82–88. DOI: 10.5176/2345-718X_4.1.135.

[bibr46-14713012241299015] McKeown J. Clarke A. Ingleton C. Repper J. (2010). Actively involving people with dementia in qualitative research. Journal of Clinical Nursing, 19(13–14), 1935–1943. DOI: 10.1111/j.1365-2702.2009.03136.x. 20920020

[bibr47-14713012241299015] McKinnon R. (2016). Epistemic injustice. Philosophy Compass, 11(8), 437–446. DOI: 10.1111/phc3.12336.

[bibr48-14713012241299015] Miller L. M. Whitlatch C. J. Lyons K. S. (2016). Shared decision-making in dementia: A review of patient and family carer involvement. Dementia, 15(5), 1141–1157. DOI: 10.1177/1471301214555542. 25370075

[bibr49-14713012241299015] Milne A. (2010). The ‘D’ word: Reflections on the relationship between stigma, discrimination and dementia. Journal of Mental Health, 19(3), 227–233. DOI: 10.3109/09638231003728166. 20441486

[bibr50-14713012241299015] Mitchell G. Dupuis S. L. Kontos P. Jonas-Simpson C. Gray J. (2020). Disrupting, dehumanising and intersecting patterns of modernity with a relational ethic of caring. International Practice Development Journal, 10(1), 1–15. DOI: 10.19043/ipdj.101.002.

[bibr51-14713012241299015] Mitchell G. J. Dupuis S. L. Kontos P. C. (2013). Dementia discourse: From imposed suffering to knowing other-wise. Journal of Applied Hermeneutics, 5, 1–19.

[bibr52-14713012241299015] Mitchell L. Burton E. Raman S. Blackman T. Jenks M. Williams K. (2003). Making the outside world dementia-friendly: Design issues and considerations. Environment and Planning B: Planning and Design, 30(4), 605–632. DOI: 10.1068/b29100.

[bibr53-14713012241299015] Novek S. Wilkinson H. (2019). Safe and inclusive research practices for qualitative research involving people with dementia: A review of key issues and strategies. Dementia, 18(3), 1042–1059. DOI: 10.1177/1471301217701274. 28350179

[bibr54-14713012241299015] O’Connor C. M. C. Liddle J. O’Reilly M. Meyer C. Cartwright J. Chisholm M. Conway E. Fielding E. Fox A. MacAndrew M. Schnitker L. Travers C. Watson K. While C. Bail K. (2022). Advocating the rights of people with dementia to contribute to research: Considerations for researchers and ethics committees. Australasian Journal on Ageing, 41(2), 309–313. DOI: 10.1111/ajag.13023. 34821448

[bibr55-14713012241299015] Pesonen H.-M. Remes A. M. Isola A. (2011). Ethical aspects of researching subjective experiences in early-stage dementia. Nursing Ethics, 18(5), 651–661. DOI: 10.1177/0969733011408046. 21893576

[bibr56-14713012241299015] Pinkert C. Köhler K. von Kutzleben M. Hochgräber I. Cavazzini C. Völz S. Palm R. Holle B. (2021). Social inclusion of people with dementia – an integrative review of theoretical frameworks, methods and findings in empirical studies. Ageing and Society, 41(4), 773–793. DOI: 10.1017/S0144686X19001338.

[bibr57-14713012241299015] Pratt R. (2002). ‘Nobody’s ever asked how I felt. In Wilkinson H. (Ed.), The perspectives of persons with dementia: Research methods and motivations (pp. 165–182). Jessica Kingsley.

[bibr58-14713012241299015] Price K. A. Hill M. R. (2021). The silence of Alzheimer’s disease: Stigma, epistemic injustice, and the inequity of those with progressive cognitive impairment. Communication Research and Practice, 7(4), 326–343. DOI: 10.1080/22041451.2021.2006113.

[bibr59-14713012241299015] Rivett E. (2017). Research involving people with dementia: A literature review. Working With Older Persons (Brighton, England), 21(2), 107–114. DOI: 10.1108/WWOP-11-2016-0033.

[bibr60-14713012241299015] Sabat S. R. (2006). Mind, meaning, and personhood in dementia: The effects of positioning. In Hughes J. C. Louw S. J. Sabat S. R. (Eds.), Dementia: Mind, meaning and the person (pp. 287–302). Oxford University Press.

[bibr61-14713012241299015] Schwertner E. Pereira J. B. Xu H. Secnik J. Winblad B. Eriksdotter M. Nägga K. Religa D. (2022). Behavioural and psychological symptoms of dementia in different dementia disorders: A large-scale study of 10,000 individuals. Journal of Alzheimer’s Disease, 87(3), 1307–1318. DOI: 10.3233/JAD-215198. PMC9198804 35491774

[bibr62-14713012241299015] Scottish Dementia Working Group Research Sub-Group, UK . (2014 Sep). Core principles for involving persons with dementia in research: Innovative practice. Dementia, 13(5), 680–685. DOI: 10.1177/1471301214533255. 24858551

[bibr63-14713012241299015] Sherratt C. Soteriou T. Evans S. (2007). Ethical issues in social research involving persons with dementia. Dementia, 6(4), 463–479. DOI: 10.1177/1471301207084365.

[bibr64-14713012241299015] Singh S. (2016). Contextualising the role of the gatekeeper in social science research. South African Journal of Bioethics and Law, 9(1), 42–46. DOI: 10.7196/SAJBL.465.

[bibr65-14713012241299015] Spencer L. (2023). Epistemic injustice in late-stage dementia: A case for non-verbal testimonial injustice. Social Epistemology, 37(1), 62–79. DOI: 10.1080/02691728.2022.2103474. 36816431 PMC9928428

[bibr66-14713012241299015] Stiggelbout A. Pieterse A. De Haes J. (2015). Shared decision making: Concepts, evidence, and practice. Patient Education and Counseling, 98(10), 1172–1179. DOI: 10.1016/j.pec.2015.06.022. 26215573

[bibr67-14713012241299015] Tanner D. (2012). Co-research with older people with dementia: Experience and reflections. Journal of Mental Health, 21(3), 296–306. DOI: 10.3109/09638237.2011.651658. 22574956

[bibr68-14713012241299015] West E. Stuckelberger A. Pautex S. Staaks J. Gysels M. (2017). Operationalising ethical challenges in dementia research – a systematic review of current evidence. Age and Ageing, 46(4), 678–687. DOI: 10.1093/ageing/afw250. 28104596

[bibr69-14713012241299015] Young J. A. Lind C. Orange J. B. Savundranayagam M. Y. (2019). Expanding current understandings of epistemic injustice and dementia: Learning from stigma theory. Journal of Aging Studies, 48, 76–84. DOI: 10.1016/j.jaging.2019.01.003. 30832933

